# Supplementary material for: Defining key concepts for mental state attribution
Source: Commun Psychol. 2024 Apr 11;2:29. doi: 10.1038/s44271-024-00077-6 (PMC11332223; doi:10.1038/s44271-024-00077-6)

Supplementary Information File:

Defining key concepts for mental state attribution

1. Supplementary Methods

Selection of the expert panel

Internationally renowned experts from different disciplines, who are actively conducting research on “mental state attribution” in the broadest sense, were contacted by the first author to join the project. The first cohort of experts was identified during a scientific exchange at the international workshop *“from self-knowledge to knowing others”* held in Brussels in November 2018, involving some of the authors of the present work. Each expert who was contacted this way to participate in the project was also encouraged to suggest other experts to exhaustively cover the whole field. Ultimately, 62 researchers were contacted. Finally, 45 agreed to take part in this collaborative work and provided input to the study. Two contributors have chosen to not be listed as authors. Researchers from our final sample originated from 12 countries (Austria, Belgium, Canada, Denmark, Germany, France, Israel, Italy, Japan, The Netherlands, The United States of America, United Kingdom) and were affiliated with 47 institutions. Experts came from a wide range of research fields (see Supplementary Table 1 below, for an illustration). Our sample of experts spans career stages and includes earlier career, mid-career and more senior researchers. The complete list of experts corresponds to the authors of the present manuscript.

Coverage of the lexicon

A list of 18 terms currently in use in the literature related to mental state attribution was generated based on Quesque and Rossetti’s (2020) terminological analysis. During the first round, participants were requested to provide three types of input to the project: First, they had to specify for each term whether they had ever encountered it (see Supplementary Figure 1 below, for an illustration). Second, they should provide a definition for each familiar

term. And finally, for each term, they had to indicate whether they thought that this term should be discontinued. After the experts submitted their definitions and recommendations, we determined the essential defining elements of their definition that were shared among the majority of experts and, on this basis, generated new definitions. During a second round of discussion, our new definitions were again sent to the experts for evaluation and amended when necessary. Terms that were identified as strict synonyms were suggested to be discontinued when less frequently used or preferred by researchers. Based on the feedback obtained during the second round of discussion, final definitions and recommendations have been proposed. Percentage of agreement was then calculated and reported for each term.

2. Supplementary Table 1. Representation of the different research fields represented in our sample. *Experts self-reported to which field(s) they identified themselves to belong. Research field labels were self-generated by each author and they could indicate as many fields as they wanted.*

Research Fields	Proportion of participants affiliated with these fields
Affective neuroscience	19.5%
Autism research	17.1%
Clinical psychology & Psychopathology	22%
Cognitive neuroscience	34.1%
Cognitive psychology	22%
Comparative psychology	2.4%
Cultural & Evolutionary psychology	4.9%
Developmental neuroscience	17.1%
Experimental psychology	19.5%
Philosophy of cognitive science	9.8%
Social neuroscience	39%
Social psychology	39%

3. Supplementary Figure 1. Proportion of experts who stated having already encountered each of the proposed terms. *Proportions are based on all experts' responses available for each question.*

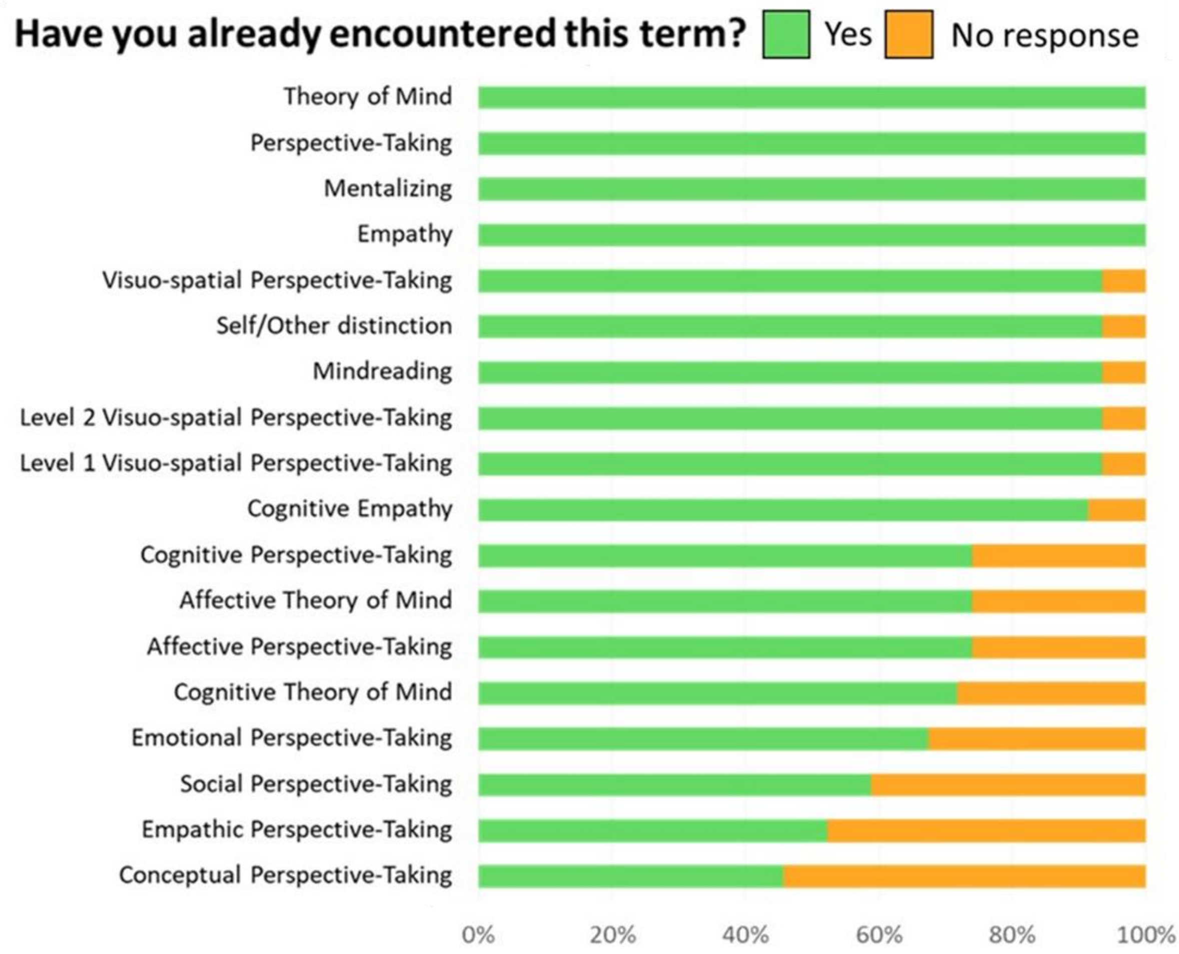

Supplement: Supplementary file 1 — Supplementary Information [file 44271_2024_77_MOESM1_ESM.pdf]
